# Syndecan-4 is required for early-stage repair responses during zebrafish heart regeneration

**DOI:** 10.1007/s11033-024-09531-4

**Published:** 2024-05-03

**Authors:** Zih-Yin Lai, Chung-Chi Yang, Po-Hsun Chen, Wei-Chen Chen, Ting-Yu Lai, Guan-Yun Lu, Chiao-Yu Yang, Ko-Ying Wang, Wei-Cen Liu, Yu-Chieh Chen, Lawrence Yu-Min Liu, Yung-Jen Chuang

**Affiliations:** 1 School of Medicine, National Tsing Hua University, Hsinchu, 300044 Taiwan, ROC; 2 Institute of Bioinformatics and Structural Biology, National Tsing Hua University, Hsinchu, 300044 Taiwan, ROC; 3 Division of Cardiovascular Medicine, Taoyuan Armed Forces General Hospital, Taoyuan City, 325208 Taiwan, ROC; 4 Cardiovascular Division, Tri-Service General Hospital, National Defense Medical Center, Taipei City, 114201 Taiwan, ROC; 5 Department of Internal Medicine, Division of Cardiology, Hsinchu MacKay Memorial Hospital, Hsinchu, 300044 Taiwan, ROC; 6 Department of Medicine, MacKay Medical College, New Taipei City, 252005 Taiwan, ROC

**Keywords:** Syndecan-4, Heart regeneration, Zebrafish, Repair response

## Abstract

**Background:**

The healing process after a myocardial infarction (MI) in humans involves complex events that replace damaged tissue with a fibrotic scar. The affected cardiac tissue may lose its function permanently. In contrast, zebrafish display a remarkable capacity for scar-free heart regeneration. Previous studies have revealed that syndecan-4 (SDC4) regulates inflammatory response and fibroblast activity following cardiac injury in higher vertebrates. However, whether and how Sdc4 regulates heart regeneration in highly regenerative zebrafish remains unknown.

**Methods and Results:**

This study showed that *sdc4* expression was differentially regulated during zebrafish heart regeneration by transcriptional analysis. Specifically, *sdc4* expression increased rapidly and transiently in the early regeneration phase upon ventricular cryoinjury. Moreover, the knockdown of *sdc4* led to a significant reduction in extracellular matrix protein deposition, immune cell accumulation, and cell proliferation at the lesion site. The expression of *tgfb1a* and *col1a1a*, as well as the protein expression of Fibronectin, were all down-regulated under *sdc4* knockdown. In addition, we verified that *sdc4* expression was required for cardiac repair in zebrafish via in vivo electrocardiogram analysis. Loss of *sdc4* expression caused an apparent pathological Q wave and ST elevation, which are signs of human MI patients.

**Conclusions:**

Our findings support that Sdc4 is required to mediate pleiotropic repair responses in the early stage of zebrafish heart regeneration.

**Supplementary Information:**

The online version contains supplementary material available at 10.1007/s11033-024-09531-4.

## Introduction

Myocardial infarction (MI), commonly known as a heart attack, is the most prevalent type of cardiovascular disease. A recent review indicated several pathophysiological processes after MI, including inflammatory response, removal of necrotic cells, angiogenesis, tissue remodeling, and fibrosis (also known as scarring) [1]. However, these wound-healing processes’ transformation and regulatory mechanisms remain unclear.

Although many therapies can protect some injured cardiomyocytes, these treatments cannot fully prevent heart exhaustion [2] due to two key issues: (1) the low regeneration ability of human myocardial cells and (2) the high prevalence of cardiac fibrosis. Insufficient cardiomyocyte regeneration can lead to chronic heart problems and failure [3]. Besides, after MI, a massive extracellular matrix (ECM) accumulates in the lesion to avoid heart rupture. Such excess deposition of fibrous tissue blocks cell regeneration and tissue remodeling [4]. This fibrous tissue deposition is due to abnormal immune responses and overly active fibroblasts, which transform into myofibroblasts, exacerbating cardiac fibrosis [4]. It is therefore crucial to identify potential therapeutic targets for reducing cardiac fibrosis and promoting cardiomyocyte regeneration after a myocardial infarction.

Zebrafish, a popular model organism for regeneration research [5], have hearts similar to human cardiac electrophysiology [6], making them a predictive cardiac electrical system model. On the other hand, zebrafish cardiac injury models have been well-established over decades [7, 8], with ventricular cryoinjury being a suitable approach to model human MI. Unlike adult mammals, adult zebrafish can fully regenerate its heart after severe ventricular damage and restore cardiac functions within months. Zebrafish heart regeneration can be subdivided into different phases. First, cardiac injury induces necrosis and inflammation for about 3 days. Next, the reparative phase begins 4 days post injury (dpi) and ends at 7 dpi. This phase is characterized by ECM deposited and cell proliferation at the injury region. After 7 dpi, cardiomyocytes proliferate until 14 dpi. After 14 dpi, regenerated cardiomyocytes replace the ECM at the scarred site. Finally, the myocardium is completely regenerated by 60 dpi [7]. Although some factors in regulating the dynamic process of zebrafish heart regeneration have been identified, understanding regulatory pathways that underlie ECM and electrical remodeling needs to be improved.

Recent studies indicate that syndecans, cell surface adhesion receptors, regulate ECM remodeling in cardiac fibrosis. Syndecans function as co-receptors for growth factors and interact with fibroblast growth factor, vascular endothelial growth factor, transforming growth factor-beta (TGF-β), and several ECM molecules. Syndecans also serve as reservoirs for ECM proteins and chemokines, regulating inflammation, wound healing, and tissue remodeling [9]. In humans, the syndecan family includes four members: syndecan 1 to 4. Among them, syndecan-4 (SDC4) mediates multiple cellular functions, including cell adhesion, migration, proliferation, endocytosis, and mechano-transduction [10].

Interestingly, a study indicated that SDC4 was abnormally elevated in acute MI patients [11]. SDC4 overexpression was also observed in damaged cardiac tissue in a mouse MI model, while scar formation and inflammatory reaction were reduced in *Sdc4* knockout MI mice [12]. Although SDC4 is known to play an essential role in cardiovascular disease, the zebrafish Sdc4 function in heart regeneration is unclear. This study hypothesized that Sdc4 regulated repair responses during early heart regeneration in zebrafish. We aimed to explore the role of Sdc4 after cryoinjury of the zebrafish heart. We hope to provide new research directions to syndecan biology and its translational application by integrating what we found.

## Materials and Methods

### Zebrafish lines and ethics statement

This study used adult zebrafish between 6 and 12 months of age, whose body lengths were 2.5–3.5 cm. The study used the wild-type AB zebrafish strain and *Tg(kdr:EGFP;lyz:DsRed)* transgenic strain. All zebrafish-use protocols in this research were reviewed and approved by the Institutional Animal Care and Use Committee of National Tsing Hua University, Hsinchu, Taiwan, R.O.C. (IRB Approval NO. 109086).

### Zebrafish anesthesia

The anesthetic protocol was followed as previously described [13]. The zebrafish were soaked in water with 60 ppm MS-222 (Sigma-Aldrich, St. Louis, MO, USA) and 60 ppm isoflurane (Baxter, Guayama, PR, USA) for 2–5 min. The anesthetic degree was checked by pressing the tail of the zebrafish.

### Zebrafish ventricular cryoinjury

The zebrafish cryoinjury experimental procedure was performed as previously described with some modifications [14]. The anesthetized zebrafish were fixed to the sponge, and the ventral side was displayed. An inverted T-shape was cut upon the heart site using cornea scissors after the scales were gently removed from the fish's chest. Then, the chest muscle was separated by tip tweezers and the pre-cool probe was placed on the tip of the ventricle for 22 s. Finally, the zebrafish were immediately transferred into a fish tank for recovery. The process is outlined in Fig. S1.

### *sdc4* siRNA and retro-orbital injection

To knockdown *sdc4* in adult zebrafish hearts, zebrafish *sdc4* siRNA (GenePharma, Shanghai, China) was used. The *sdc4* siRNA sequence is shown in Table S1. The following protocol was performed as previously described with some modifications [15]. For preparing the *sdc4* siRNA/SilenceMag nanoparticle mix, 40 μL of 10 nM *sdc4* siRNA was mixed with 1.1 μL SilenceMag agent (OZ Bioscience, Marseille, France). The experimental procedure and the knockdown efficiency of *sdc4* siRNA are shown in Fig. S2.

The retro-orbital injection was used to deliver siRNA-loaded magneto-nanoparticles into the zebrafish bloodstream. The anesthetized zebrafish were placed on the Petri dish and covered with wetted tissue paper. The needle was inserted into the zebrafish eye socket with 1–2 mm depth by a 7 o'clock position and at a 45-degree angle and injected with 2 μL *sdc4* siRNA/SilenceMag nanoparticle mix into the zebrafish blood vessel. The treated zebrafish were then transferred into a recovery tank with fresh water.

### RNA isolation and real-time quantitative PCR analysis

The hearts of the zebrafish were dissected and soaked in phosphate-buffered saline (PBS) to remove blood, bulbus arteriosus, and atrium. Total RNA from the ground ventricles was extracted by TRIzol reagent (Invitrogen, Carlsbad, CA, USA) according to the manufacturer’s protocol. The genome DNA was removed by DNase I (Invitrogen, Carlsbad, CA, USA). The cDNA was synthesized by Transcriptor First Strand cDNA Synthesis Kit (Roche, Mannheim, Germany). The *Power* SYBR^®^ Green PCR Master Mix (Applied Biosystems, Warrington, UK) was used for real-time quantitative PCR on ABI StepOnePlus™ Real-Time PCR System. The sequence of the primers used is provided in Table S2.

### Cryosection and acid fuchsin orange G (AFOG) staining

All zebrafish hearts were incubated in 10 mM glycine in 70% ethanol overnight at 4 °C and then incubated in 30% sucrose with PBS overnight. The hearts were fixed in Tissue-Tek^®^ O.C.T. Compound (Sakura Finetek USA, Torrance, CA, USA) at – 80 °C. The hearts were sectioned with a cryostat (CM3050S; Leica Biosystems, Deer Park, IL, USA) at 12 μm thickness for the staining.

For AFOG staining, the sections were fixed in Bouin’s solution (Sigma-Aldrich, St. Louis, MO, USA) for 1 h and rinsed with distilled water. Then, the sections were incubated in 1% phosphomolybdic acid (Sigma-Aldrich, St. Louis, MO, USA) for 5 min, rinsed with distilled water, and stained with 1% acid fuchsin (Sigma-Aldrich, St. Louis, MO, USA) for 5 min. The sections were rinsed with distilled water again and then stained with a dye mix containing 2% orange G (Sigma-Aldrich, St. Louis, MO, USA), 0.5% aniline blue (Sigma-Aldrich, St. Louis, MO, USA), and 1% phosphotungstic acid (Sigma-Aldrich, St. Louis, MO, USA) for 15 min. Stained sections were dehydrated with ethanol and xylene and mounted with the mounting reagent (Fisher Scientific, Fair Lawn, NJ, USA).

### Immunofluorescence staining

The slides of the heart section were washed in 0.1% WPBS buffer (PBS with 0.1% Tween 20). The blocking reagent was 2% bovine serum albumin in 0.1% WPBS. The slides were blocked for 1 h at room temperature. Then, the slides were incubated with primary antibodies at 4°C overnight. After the slides were washed in WPBS, the slides were hybridized with a secondary antibody at room temperature for 1 h. Finally, the slides were stained by Hoechst for 15 min, washed in WPBS, and mounted in ProLong™ Gold antifade reagent (Invitrogen, Eugene, OR, USA). Images were captured by using a confocal microscope (LSM 510 META; Carl Zeiss, Jena, Germany) or an inverted fluorescent microscope (TE2000E; Nikon, Kanagawa, Japan). The quantitative analyses of fluorescence images were processed using the Image-pro plus AMS software (Media Cybernetics, Bethesda, MD, USA).

The primary antibodies were anti-PCNA (AS-55421; AnaSpec, Fremont, CA, USA), anti-myosin heavy chain (MF-20; DSHB, Iowa City, IA, USA), anti-Fibronectin (F3648; Sigma-Aldrich, St. Louis, MO, USA) and anti-Syndecan-4 (X2-Q0JY12; AbInsure, Berkeley Heights, NJ, USA). The anti-rabbit DyLight 488 and anti-mouse DyLight 549 secondary antibodies were obtained from Jackson ImmunoResearch Laboratories (West Grove, PA, USA). Nuclei were stained with Hoechst 33342 (Invitrogen).

### Zebrafish electrocardiography (ECG) recording

The ECG procedure was performed as previously described [16]. After anesthetizing the zebrafish, as mentioned above, the zebrafish were placed into a Y-shaped cleft in a wet sponge to maintain the ventral side up. During the real-time ECG recording, two-needle electrode probes (*i.e.*, one pectoral electrode and one abdominal electrode) were gently inserted into the zebrafish body, and the third needle electrode probe (*i.e.*, the grounding electrode) was inserted into the sponge as a reference electrode. After recording, the zebrafish were then immediately transferred to a clean recovery water tank after recording.

### Statistical analysis

All experiment data were expressed as the mean ± standard error of the mean (SEM). Statistical analysis was performed by GraphPad Prism version 9 program (San Diego, CA, USA) using Student’s *t*-test. *p* < 0.05 was considered statistically significant.

## Results

### Differential *sdc4/Sdc4* expression during zebrafish heart regeneration and cardiac repair in mice was observed

To investigate the role of *sdc4* in cardiac repair and regeneration, we analyzed published microarray datasets from adult zebrafish subjected to ventricular cryoinjury or ventricular amputation, as well as mice subjected to LAD (left anterior descending) coronary artery occlusion (NCBI GEO accession number: GSE94617, GSE72348, and GDS2331). The results were then validated using real-time quantitative PCR (RT-qPCR).

**Fig. 1:**
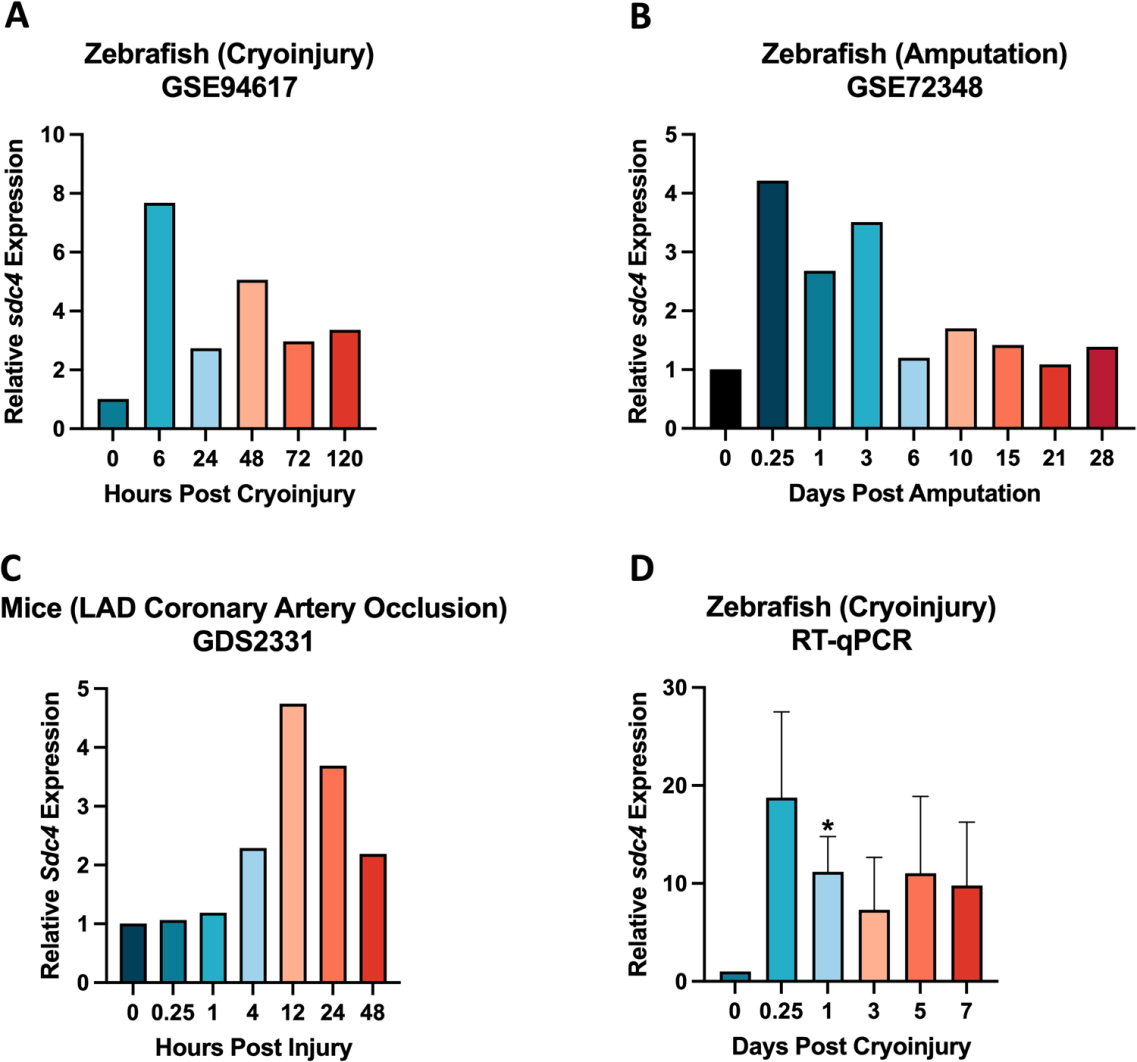
*sdc4/Sdc4* expression profile analyses between zebrafish and mice after heart injury. **A** The zebrafish ventricles were collected at different time points after cryoinjury for microarray analysis: 0, 6, 24, 48, 72 and 120 hpci. (NCBI GEO accession number: GSE94617) (hpci: hours post cryoinjury). **B** The zebrafish ventricles were collected at different time points after ventricular resection for microarray analysis: 0, 0.25, 1, 3, 6, 10, 15, 21 and 28 dpa. (NCBI GEO accession number: GSE72348) (dpa: days post amputation). **C** The mice ventricles were collected at different time points after injury for microarray analysis: 0, 0.25, 1, 4, 12, 24 and 48 hpi. (NCBI GEO accession number: GDS2331) (hpi: hours post injury). **D** The zebrafish ventricles were collected at different time points after ventricular cryoinjury for real-time quantitative PCR (RT-qPCR): 0, 0.25, 1, 3, 5 and 7 dpci. (*p < 0.05 vs. 0 dpci; n = 6 per group) (dpci: days post cryoinjury)

In the zebrafish microarray datasets, *sdc4* expression rapidly increased in the early stage of heart regeneration following either cryoinjury or amputation after heart injury (Fig. 1A, B). Notably, *sdc4* gene expression peaked at 6 h post injury (hpi) in both datasets, with a 7.68-fold increase in the cryoinjury dataset (Fig. 1A) and a 4.21-fold increase in the amputation dataset (Fig. 1B). In the mouse microarray dataset of acute cardiac injury response (Fig. 1C), *Sdc4* showed a transient increase after LAD coronary artery occlusion, with a 2.29-fold and 4.74-fold increase at 4 and 12 hpi, respectively. These published microarray datasets suggest that *sdc4/Sdc4* plays a role in the early stage of heart injury and repair.

We performed RT-qPCR analysis to validate the microarray findings during the early stage of zebrafish heart regeneration following ventricular injury (Fig. 1D). Consistent with our expectations, *sdc4* gene expression increased immediately after cryoinjury, peaking at 18.76-fold at 6 h and then steadily decreasing to tenfold at subsequent time points. These data suggest that *sdc4* expression rapidly increase in the early stage of heart regeneration and may play a role in this process.

### Inhibition of *sdc4* reduced collagen deposition during zebrafish heart regeneration

A previous study indicated that the *Sdc4*-deficient mice exhibited reduced ECM protein deposition in the damaged region following myocardial infarction [12], suggesting that SDC4 may regulate scar formation in vertebrates. To determine whether Sdc4 regulates scar formation during zebrafish heart regeneration, we monitored scar formation using histological analysis at 3- and 7-days post cryoinjury (dpci). Histological sections stained with acid fuchsin orange G (AFOG) showed normal myocardium tissue in light orange, the collagen-rich region in blue, and fibrin in red.

**Fig. 2:**
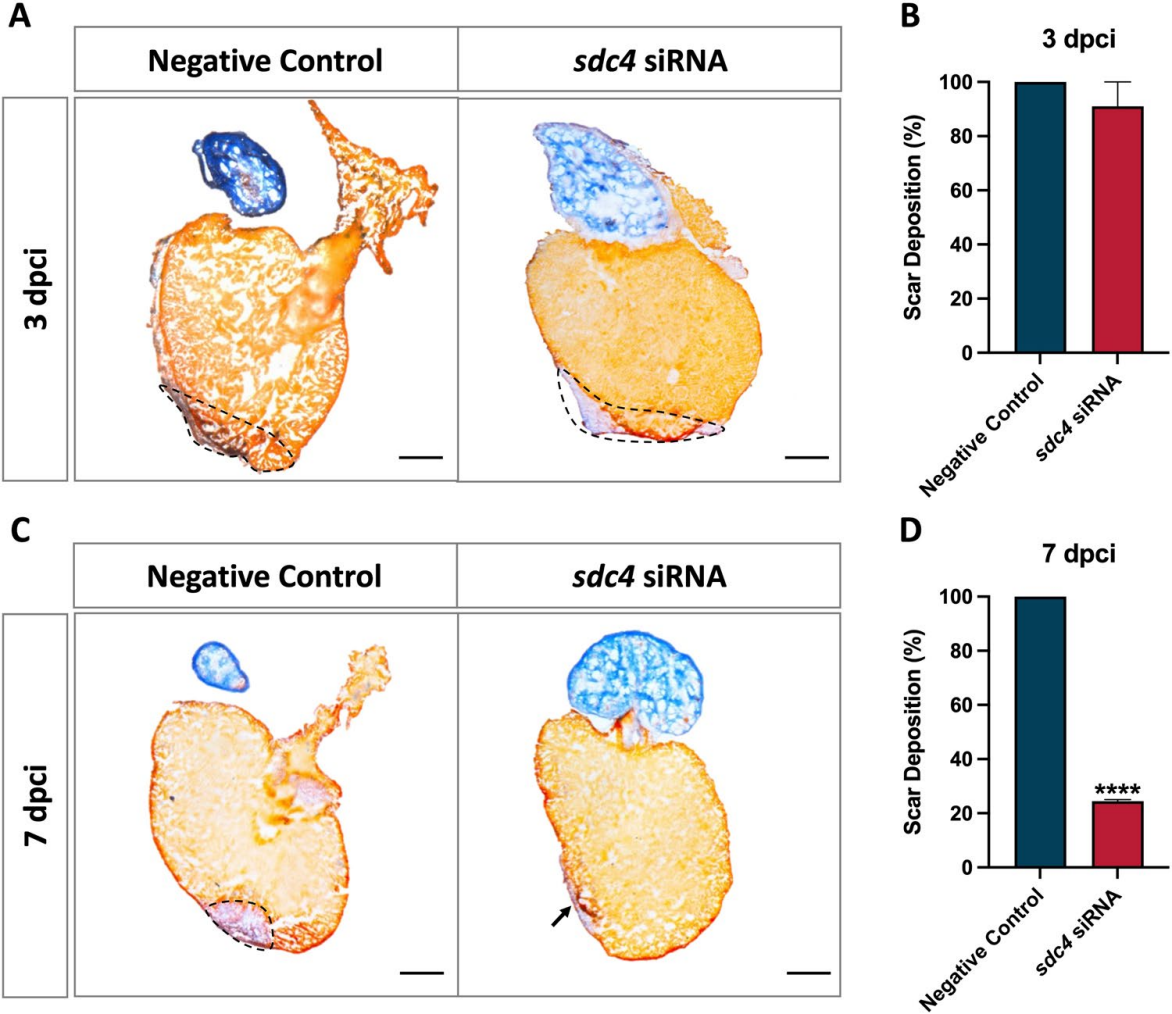
Knockdown of *sdc4* expression decreased the scar deposition of zebrafish hearts. Acid fuchsin orange G (AFOG) staining of heart sections was used to visualize fibrin (red), myocardium (light orange), and collagen (blue). Dashed line: post-cryoinjury area. Data showed scar deposition both in the negative control group and *sdc4* siRNA group at 3 dpci (**A**) and 7 dpci (**C**). The hearts of the negative control group contained abundant collagen at the injury area at 7 dpci. However, in the *sdc4* siRNA group, the scar was nearly completely resolved and replaced with new muscle. The statistical results showed that the scar deposition percentage of zebrafish hearts after cryoinjury did not change at 3 dpci (**B**) but significantly reduced in the *sdc4* siRNA group compared to the negative control group at 7 dpci (**D**). Scale bar: 200 μm (****p < 0.001 vs. negative control; n = 3–5 per group) (dpci: days post cryoinjury)

Our data showed that the expression levels of collagen deposition and fibrin-rich blood clots in the ventricular apex were similar in the negative control and *sdc4* knockdown groups at 3 dpci (Fig. 2A, B). However, at 7 dpci, abundant collagen deposition was still observed at the injury site in the negative control group, while only slight collagen staining was found in the *sdc4* knockdown group (Fig. 2C). Quantitatively, collagen-rich deposition was significantly reduced by 75% in the siRNA group compared to the negative control group (Fig. 2D). These findings suggest that Sdc4 is required for collagen formation during zebrafish heart regeneration.

### Sdc4-dependent recruitment of innate immune cells to the ventricular lesion site

Mammalian SDC4 has been shown to influence the inflammatory response, particularly with regard to immune cell accumulation in mice studies [17]. However, it is not known whether Sdc4 regulates the inflammatory response during zebrafish heart regeneration. To investigate this, we used a double-transgenic zebrafish line (*kdr:EGFP;lyz:DsRed*) to monitor the innate immune response in the early phase of zebrafish cardiac repair. This line labeled endothelial cells and myeloid cells (*i.e.*, macrophages, neutrophils, and other lysozyme expressing granulocytes).

**Fig. 3:**
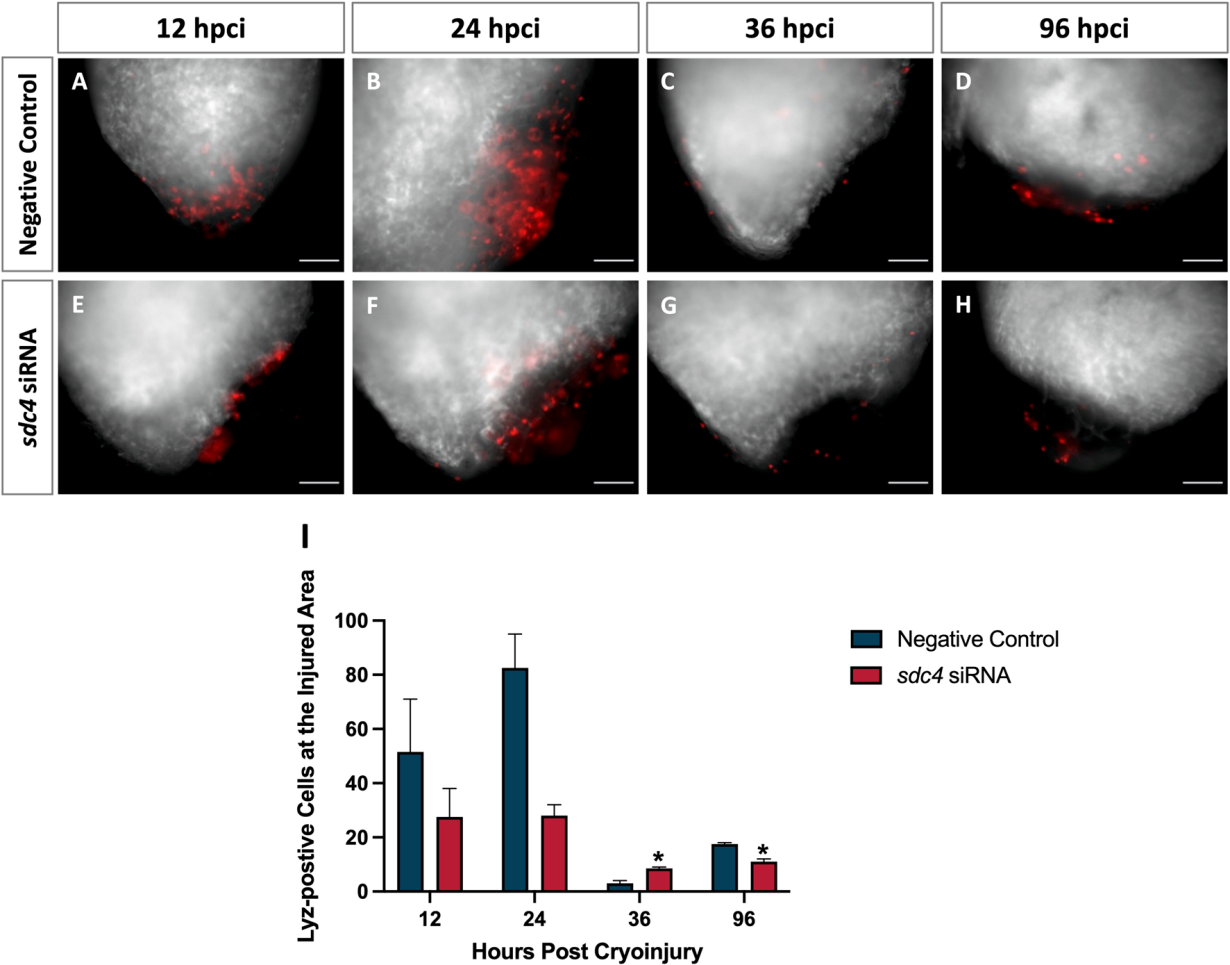
Inhibition of *sdc4* reduced the recruitment of innate immune cells to the injury site after cryoinjury. **A–H** The whole mount view of post-cryoinjury ventricles at 12, 24, 36 and 96 hpci. White: endothelial cells (*kdr:EGFP*); Red: myeloid cells (*lyz:DsRed*). **I** Quantitative analysis showed the cell numbers of myeloid cells at the injury site at each time point. The silence of *sdc4* slightly decreased the aggregation of myeloid cells at the injury site at 12 and 24 hpci and significantly reduced it at 96 hpci. Scale bar: 100 μm (*p < 0.05 vs. negative control) (hpci: hours post cryoinjury)

In the negative control group, clusters of myeloid cells were observed at the injury site at 12 h post cryoinjury (hpci) (Fig. 3A) and a 1.6-fold increase at 24 hpci (Fig. 3B). Interestingly, myeloid cell signals dramatically diminished at 36 hpci (Fig. 3C) and 96 hpci (Fig. 3D). In contrast, in the *sdc4* knockdown group, only half as many myeloid cells were observed at 12 hpci compared to the negative control (Fig. 3E), and myeloid cell counts remained low throughout the observation period from 24 to 96 hpci (Fig. 3F–H). Quantitative analysis was shown in Fig. 3I. Knockdown of *sdc4* slightly decreased myeloid cell aggregation at the injury site at 12 and 24 hpci and significantly reduced at 96 hpci.

The results suggest that Sdc4 may be required for myeloid cells migration and retention at the injury site, leading us to hypothesize that Sdc4 may contribute to the removal of apoptotic cells during the early phase of zebrafish cardiac repair.

### Knockdown of *sdc4* impaired the onset of injury-induced cell proliferation

A previous study showed that SDC4 was required to maintain the proliferation ability of skeletal muscle cells [18]. However, it is not known whether SDC4 regulates cell proliferation and migration during heart regeneration. To investigate this, we examined cell proliferation at the ventricular injury site in zebrafish with *sdc4* knockdown.

**Fig. 4:**
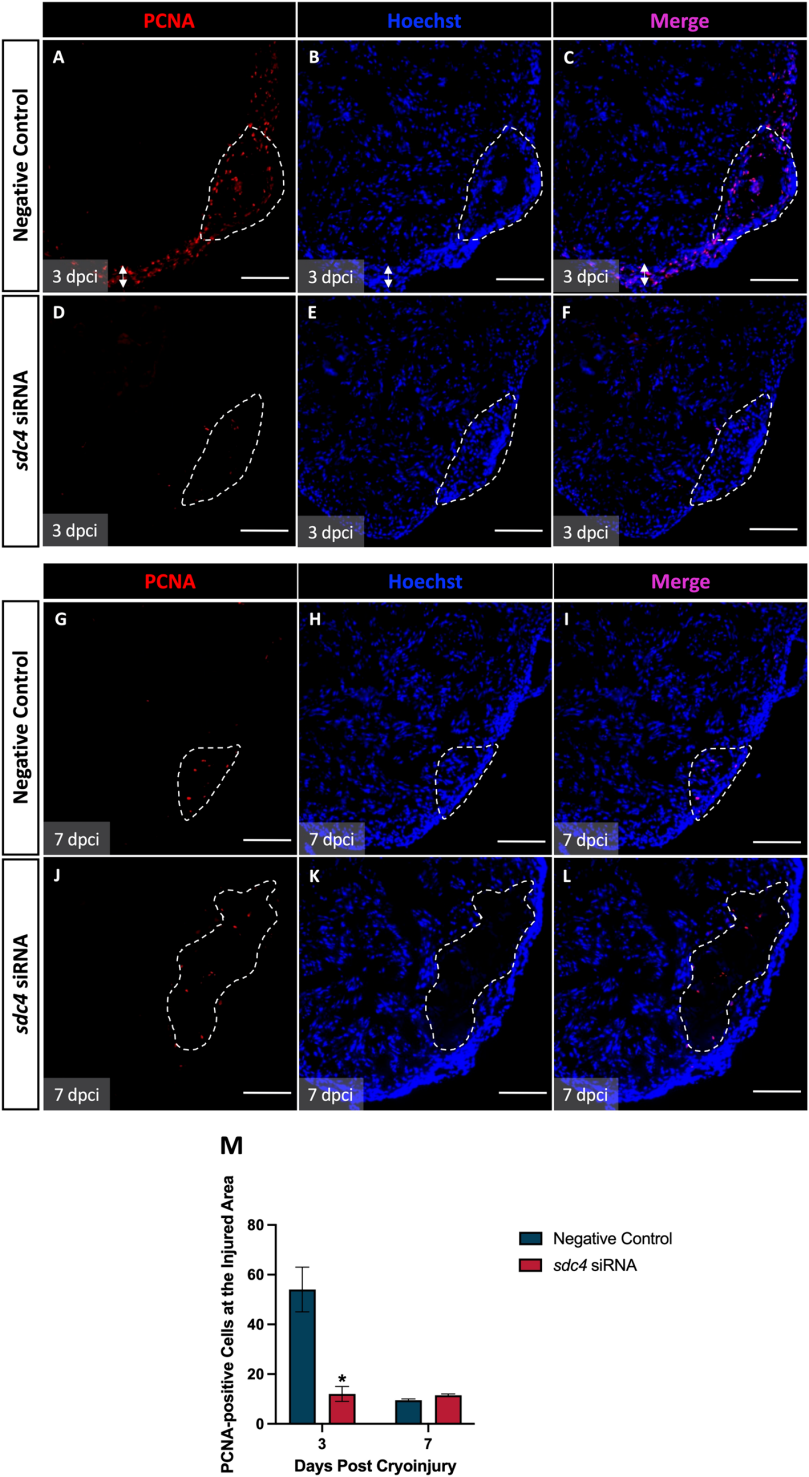
*sdc4* inhibition resulted in a decrease in cell proliferation at the injury site. Expression patterns of PCNA (red) and Hoechst (blue) immunostaining were analyzed after cryoinjury. PCNA (proliferating cell nuclear antigen) was used as a marker for cell proliferation, and Hoechst was used to label nuclei. Dashed line: injury site. Double-headed arrow: compact myocardium. **A–F** The PCNA signals of the negative control group were significantly higher than the *sdc4* siRNA group at 3 dpci. **G–L** At 7 dpci, the expression levels of PCNA were the same at the injury site in both groups. Moreover, the Hoechst signals of the *sdc4* siRNA group were weaker at the injury site than the negative control group. **M** The statistics indicated the cell numbers of PCNA-positive cells at the injury site. Knockdown of *sdc4* significantly reduced the cell proliferation at the injury site at 3 dpci, but no significant difference compared to the negative control at 7 dpci. Scale bar: 100 μm (*p < 0.05 vs. negative control; n = 2–4 per group) (dpci: days post cryoinjury)

We used proliferative cell nuclear antigen (PCNA) immunostaining to track cell proliferation. At 3 dpci, the negative control group showed an increase in PCNA signals at the injury site and along the compact myocardium (Fig. 4A–C), while injury-induced cell proliferation was significantly reduced in the *sdc4* knockdown group (Fig. 4D–F). At 7 dpci, both negative control and *sdc4* knockdown groups showed weak PCNA levels at the injury site (Fig. 4G–L), with sparse PCNA expression only observed at the injury site and not in the surrounding compact myocardium. Additionally, weak Hoechst staining was observed at the injury site in the *sdc4* knockdown group (Fig. 4J–L). Statistical analysis revealed that *sdc4* knockdown led to a significant reduction in cell proliferation at the injury site at 3 dpci, but no significant difference was observed compared to the negative control group at 7 dpci (Fig. 4M). Interestingly, we observed that the PCNA-positive cells at the injury site were not cardiomyocytes in our preliminary analysis (Fig. S3). These data suggest that *sdc4* knockdown can delay the onset of post-injury cell proliferation at 3 dpci.

### Sdc4 stimulated the expression of marker genes involved in extracellular matrix (ECM) remodeling

**Fig. 5:**
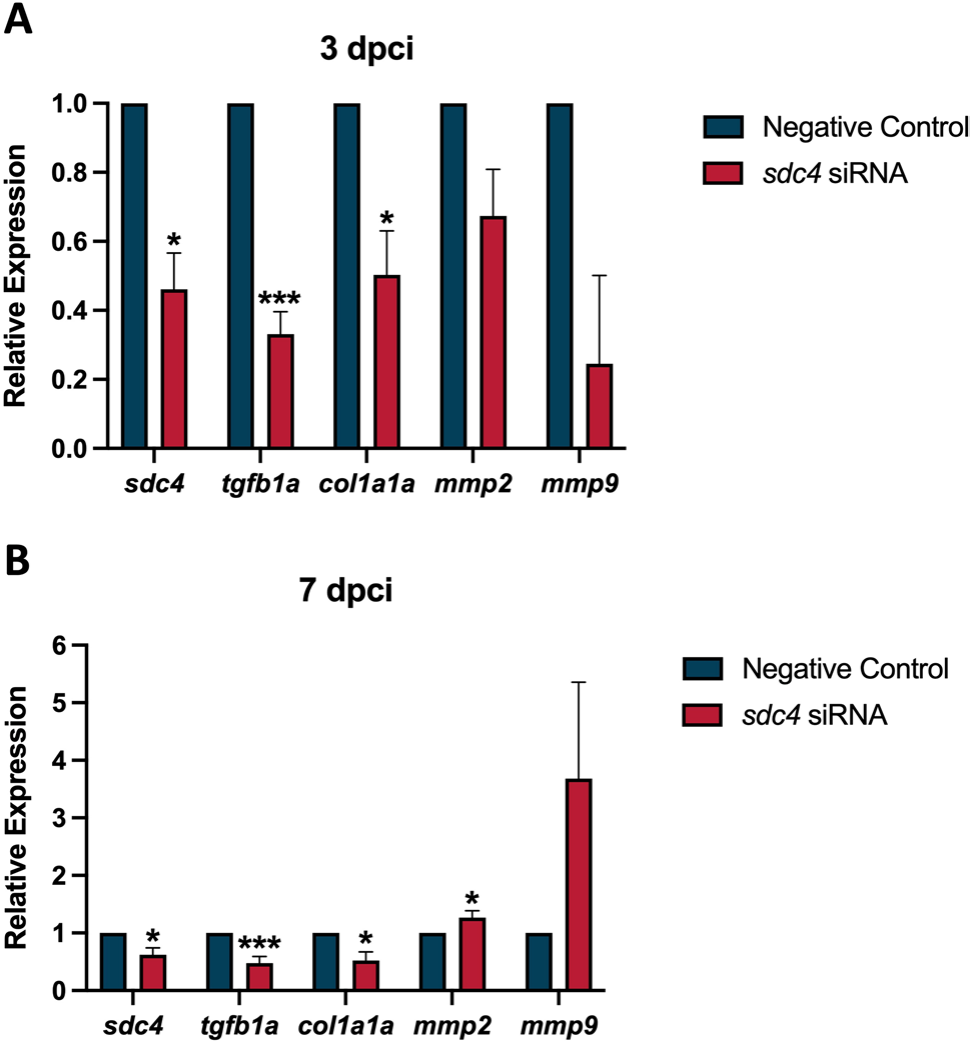
*sdc4* expression affected ECM-associated marker genes during the cardiac repair. Extracellular matrix (ECM)-associated marker genes: *tgfb1a, col1a1a, mmp2,* and *mmp9* were examined by real-time quantitative PCR with or without *sdc4* siRNA treatment at 3 and 7 dpci. **A** After treating *sdc4* siRNA**,** the *sdc4* gene expression was down-regulated as expected. Besides, *tgfb1a and col1a1a* were significantly down-regulated after *sdc4* siRNA treatment at 3 dpci. **B** At 7 dpci, expression of *sdc4, tgfb1a, and col1a1a* were all significantly reduced in the *sdc4* siRNA group. However, mRNA expression of *mmp2* was significantly up-regulated after the *sdc4* knockdown (*p < 0.05 vs. negative control; ***p < 0.005 vs. negative control; n = 8 per group) (dpci: days post cryoinjury)

To determine whether Sdc4 regulates scar formation during zebrafish heart regeneration, we analyzed the expression profiles of specific ECM-associated marker genes, including the regenerative driver (*tgfb1a*), the ECM protein (*col1a1a*), and two ECM proteinases (*mmp2* and *mmp9*) (Fig. 5).

At 3 dpci (Fig. 5A), *sdc4* was significantly reduced to 46% of negative control level upon siRNA knockdown, confirming previous assay data (Fig. S2D). We also observed the expected decrease in *tgfb1a* and *col1a1a* gene expression, which are known to differentiate fibroblasts into myofibroblasts and promote ECM synthesis [19]. The *tgfb1a* was dramatically reduced to 33% of negative control level under siRNA treatment, while *col1a1a* expression was down-regulated to 50% of negative control level following *sdc4* knockdown. The mRNA levels of *mmp2* and *mmp9* were also slightly down-regulated upon *sdc4* knockdown at 3 dpci, with *mmp2* reduced to 67% and *mmp9* reduced to 25% of negative control level (no significant difference).

At 7 dpci (Fig. 5B), *sdc4* expression remained down-regulated as expected. The expression levels of *tgfb1a* and *col1a1a* showed a slight increase compared to those observed at 3 dpci, with *tgfb1a* increasing to 48% and *col1a1a* increasing to 52% of negative control level. Strikingly, *mmp2* expression exhibited a significant 1.27-fold increase, while *mmp9* was up-regulated by 3.68-fold following *sdc4* knockdown. In summary, these data suggest that Sdc4 is primarily required for ECM production in the early stage of zebrafish heart regeneration.

### Inhibition of *sdc4* impaired the production of ECM protein

The cardiac ECM is composed of a variety of proteins, including structural proteins such as collagen and adhesive protein such as Fibronectin. Recent studies have shown that SDC4 is up-regulated following tissue injury and that its heparan sulfate chains bind to Fibronectin and integrin, collectively inducing the formation of focal adhesions and stress fibers [20]. Fibronectin provides a scaffold for cell adhesion and is extensively expressed during the wound healing process. Our additional analysis revealed a correlation between Sdc4 and Fibronectin expression at the cryoinjury site (Fig. S4). To determine the potential effect of Sdc4 on ECM, we investigated Fibronectin levels following *sdc4* knockdown using immunofluorescence staining.

**Fig. 6:**
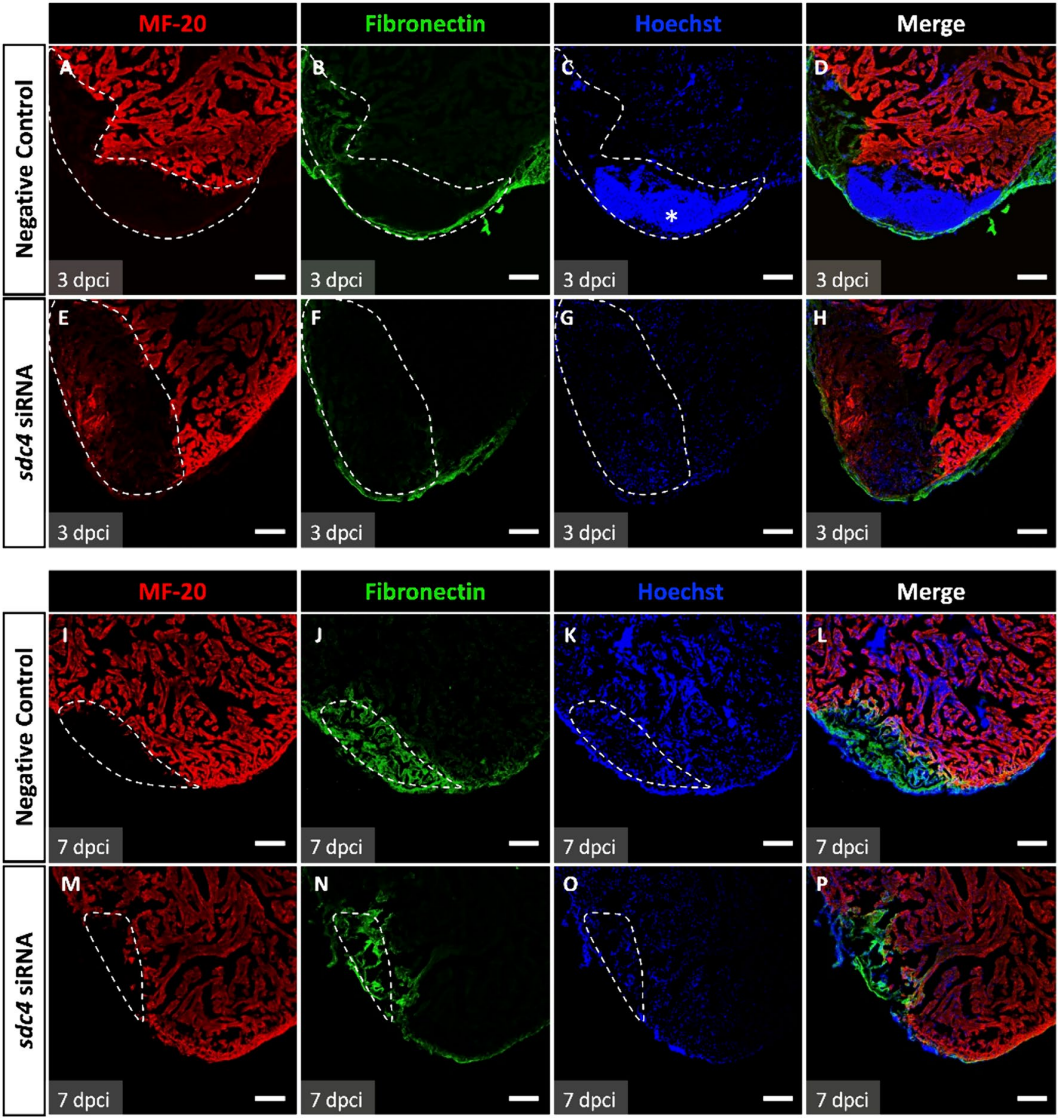
*sdc4* knockdown inhibited Fibronectin expression at the injury site. Immunofluorescence staining of heart sections after cryoinjury was performed to visualize MF-20 (red), Fibronectin (green), and Hoechst (blue). MF-20 is a myosin heavy chain labeled myocardium. Hoechst labeled nuclei. Dashed line: injury site. **A–D** In negative control group at 3 dpci, MF-20 signals at the injury site were absence. Fibronectin signals were observed in the margin of the infarct area and partially expressed at the injury site. The large DNA fragments were detected at the injury site labeled by Hoechst. (*labeled the strong signals of Hoechst). **E–H** In the *sdc4* siRNA group at 3 dpci, the signals of Fibronectin were nearly undetectable. Also, the Hoechst signals were absent at the injury area. **I–L** In the negative control group at 7 dpci, the Fibronectin signals were more highly expressed than 3 dpci of the negative control group at the injury area. **M–P** In the *sdc4* siRNA group at 7 dpci, the reduction of Fibronectin signals was observed compared to the negative control group. Scale bar: 100 μm (dpci: days post cryoinjury)

At 3 dpci (Fig. 6A–H), large infarcts devoid of cardiomyocytes were observed in both groups, as outlined by the dashed lines and labeled by anti-myosin heavy chain (anti-MF-20) (Fig. 6A, E). The infarct area contained more Fibronectin signals in the negative control group than in the siRNA group (Fig. 6B, F). Interestingly, strong DNA signals representing blood cells were detected by Hoechst staining within the lesion site in the negative control group but not in the siRNA group (Fig. 6C, G). At 7 dpci (Fig. 6I–P), the infarct area (dashed lines) showed signs of reduction (Fig. 6I, M). Strikingly, far fewer Fibronectin signals were detected at the lesion site in the siRNA group compared to the negative control group (Fig. 6J, N), suggesting impaired Fibronectin production following *sdc4* knockdown. These findings indicate that Sdc4 is required for Fibronectin production.

### Pathological Q wave and ST elevation were detected after *the sdc4* knockdown

Macrophages have been shown to play a role in cardiac conduction [21]. However, it is not known whether macrophages affect electrical conduction during zebrafish heart regeneration. Since our results demonstrated that Sdc4 was associated with myeloid cell aggregation following cryoinjury, we investigated cardiac conduction recovery during regeneration in zebrafish using electrocardiography (ECG), with and without *sdc4* siRNA treatment.

**Fig. 7:**
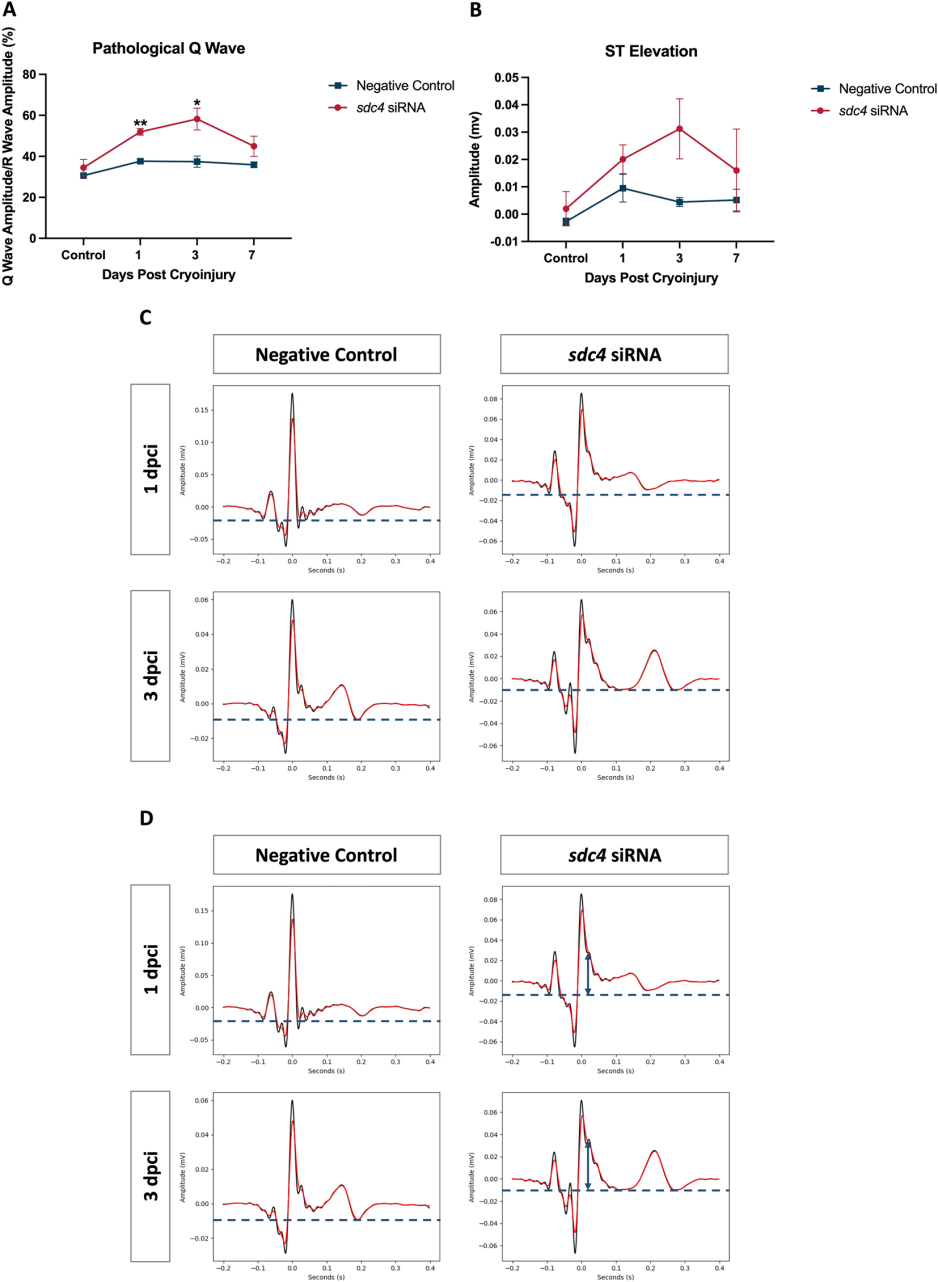
Pathological Q wave and ST elevation were observed after *sdc4* siRNA treatment. The zebrafish electrocardiography (ECG) with or without *sdc4* siRNA treatment was analyzed at control, 1, 3, and 7 dpci after cryoinjury. Two ECG signs of myocardial infarction (MI) were analyzed: pathological Q wave and ST elevation. **A, B** *sdc4* knockdown group showed significantly augmented pathological Q wave and slight ST elevation compared to the negative control group, which suggested sustained cardiac conduction abnormalities. **C, D** Representative electrocardiogram figures of the pathological Q wave and ST elevation at 1 and 3 dpci in the negative control group and *sdc4* siRNA group. (*p < 0.05 vs. negative control; **p < 0.01 vs. negative control; n = 3–7 per group) (dpci: days post cryoinjury)

We used our well-established adult zebrafish ECG system to record ECG at control, 1, 3, and 7 dpci (Fig. 7), and our analysis also referred to well-documented ECG patterns in human myocardial infarction (MI). Two major MI patterns in ECG are pathological Q waves and ST segment elevation [22]. A pathological Q wave in MI is classified as being 1/3 larger than the R wave in humans. Once a pathological Q wave is observed, it usually indicates irreversible injury. On the other hand, ST elevation occurs when the ST segment appears higher than the baseline of the PQ segment. This pattern typically reflects acute thrombotic coronary occlusion due to conductivity changes in the ischemic area.

Following *sdc4* knockdown, we observed significant pathological Q waves at 1 and 3 dpci that decreased at 7 dpci. Compared to the negative control group, the extent of pathological Q waves (as shown by the Q/R amplitude ratio) in the *sdc4* siRNA group dramatically increased to 51.98% at 1 dpci, then to 58.20% at 3 dpci, and decreased to 44.95% at 7 dpci (Fig. 7A, C). Furthermore, the ST segment was slightly elevated at 1 and 3 dpci and decreased at 7 dpci. Specifically, the ST segment elevated to 0.020 ± 0.005 mV, 0.031 ± 0.011 mV, and 0.016 ± 0.015 mV at 1, 3, and 7 dpci in the *sdc4* siRNA group, respectively (Fig. 7B). The ECG wave showed that *sdc4* knockdown resulted in significant ST segment elevation at 1 and 3 dpci compared to the negative control group (Fig. 7D). These findings suggest that reduced *sdc4* expression can disrupt the recovery of cardiac conductivity and repair progression during zebrafish heart regeneration.

## Discussion

This study examined the role of Sdc4 in zebrafish heart regeneration. We first analyzed the expression profile of *sdc4* after ventricular cryoinjury and investigated the impact of *sdc4* knockdown on heart regeneration. We also performed an ECG analysis to assess the effect of *sdc4* down-regulation on ventricular electrical remodeling, including the presence of pathological Q wave and ST elevation, which are significant indicators in Human MI. Our findings showed that Sdc4 could mediate immune cell movement, ECM proteins production, cell proliferation, heart contractility, and overall progress in the early zebrafish heart repair. These results suggest that Sdc4 has pleiotropic effects during zebrafish regeneration.

Compared to adult mice, which have limited heart regeneration capacity, Sdc4 displays similar yet distinct effects in regulating cardiac repair. Since it has been reported that the zebrafish can lose the ability to undertake scar-free cardiac healing when innate immune cells *(i.e.*, macrophages, neutrophils) are blocked from infiltrating into the damaged site [23], this raises the question of how Sdc4 regulates immune responses. By examining the temporal profiles of the immune cell recruitment (Fig. 3), we presume that upon injury, the myeloid cells migrated into the damaged tissue within 36 h might be neutrophils, and arrived by 96 h might be macrophages. Therefore, the timing of *sdc4*’s dynamic expression was critical, as its knockdown could delay and reduce neutrophils and macrophages recruitment. It is conceivable to speculate that the disruption of Sdc4, especially during the early inflammatory responses, could delay the secretion of cytokines, growth factors, and tissue remolding enzymes, all essential for the timely cardiac regeneration. And we speculate that the PCNA-positive cells (Fig. 4) at the injury site are these aggregated immune cells after their expansion during the initial inflammatory phase [24].

Previous study indicates that zebrafish heart regeneration commences in three phases: the inflammatory phase, the reparative phase, and the regeneration phase [25]. This study focuses on Sdc4’s role in the inflammatory and reparative phases of zebrafish heart regeneration, where fibroblast accumulation and excessive ECM protein deposition can distort heart architecture and function. ECM remodeling is critical for maintaining myocardial tension and elasticity during cardiac healing, and its regulation can prevent post-MI heart failure. In non-regenerative mice, SDC4 has been linked to ECM remodeling and cardiac fibroblast differentiation in response to mechanical stress. However, its role in highly regenerative zebrafish is unclear. In this study, Sdc4 regulated ECM remodeling in the zebrafish heart following cryoinjury (Fig. 5). The expression levels of *tgfb1a* and *col1a1a* were down-regulated under *sdc4* siRNA treatment at 3 dpci; however, we found that the expression of ECM cleaving enzymes, *mmp2* and *mmp9*, remained mostly unchanged at this time point. Interestingly, *mmp2* expression exhibited a significant increase at 7 dpci. It is also noteworthy that the temporal expression pattern analysis of other related genes showed that *tgfb1a* and *col1a1a* expression were continuously suppressed until 7 dpci.

Since TGF-β is known to regulate collagen synthesis and fibrosis in several diseases, we proposed that the observed decrease of *col1a1a* expression via the Sdc4-dependent regulation during early zebrafish heart regeneration might be linked to TGF-β-related pathways. This was supported by reduced Fibronectin expression in damaged tissue and compact myocardium under *sdc4* knockdown (Fig. 6). In addition, it has been shown that increased Fibronectin can stimulate the activation of epicardial derived cells' (EPDCs) into fibroblasts and myofibroblasts [26], or promote cell migration and proliferation [27]. It is conceivable that Sdc4 might also affect cell proliferation during heart regeneration by the Fibronectin-dependent pathway. The EPDCs in adult mice cannot commence scar-free healing after cardiac injury, while neonatal mice have a zebrafish-like regeneration capacity [28]. Thus, how Sdc4 regulates the differential responses stimulated by TGF-β between adult and neonatal mice shall be an interesting question to answer in future studies.

The ECG has been used to monitor the electrophysiological function of the heart during zebrafish heart regeneration. In this study, we adapted and improved the ECG test to record pathological Q wave and the ST elevation, which were not clearly presented in prior zebrafish-based studies (Fig. 7). The improvement mainly arose from how the ECG probes were placed, effectively reducing the electrical signal noises generated by gill movement and chest muscle contraction. Therefore, the zebrafish ECG method shown in the study has high potential for using in large-scale MI drug screening.

Comparing regenerative and non-regenerative hearts can inform treatment strategies for human heart diseases. In healthy adult humans, ventricular blood pressure is maintained at 120 mmHg, while in adult zebrafish, it is estimated to be only 2.5 mmHg [29]. Mammals deposit thick collagen layers to strengthen the ventricular wall against high blood pressure [30], whereas zebrafish generate a thin, loose collagen-rich scar during early heart regeneration [31], making the scarring easier to resolve.

Our findings raise critical questions about the mechanism involved in heart regeneration in zebrafish and humans. For example, while *sdc4* knockdown can reduce scar formation, does it compromise the strength of the new cardiac muscle? Is Sdc4 involved in balancing mechanical strength and contraction function? What are the specific types of immune cells involved in the heart’s response to cryoinjury? Further analysis of Sdc4 and ECM-related cell interaction is needed to provide insights into heart regeneration biology.

In conclusion, this study highlights that *sdc4* knockdown results in compromised repair responses, altering ECM remodeling after ventricular injury. Our findings help further clarify the complex mechanisms involved in zebrafish heart regeneration, broadening our understanding of regeneration biology.

## Supplementary Information

Below is the link to the electronic supplementary material.

Supplementary file1 (DOCX 5892 KB)

## Data Availability

The manuscript contains all data supporting the reported results.

## Abbreviations

AFOG: Acid fuchsin orange G
Dpi: Days post injury
Dpci: Days post cryoinjury
Dpa: Days post amputation
ECG: Electrocardiogram/electrocardiography
ECM: Extracellular matrix
EPDCs: Epicardial derived cells
Hpi: Hours post injury
Hpci: Hours post cryoinjury
LAD: Left anterior descending
MI: Myocardial infarction
PBS: Phosphate-buffered saline
PCNA: Proliferative cell nuclear antigen
RT-qPCR: Real-time quantitative PCR
SDC4: Syndecan-4
TGF-β: Transforming growth factor-beta

## Declarations

### Animal studies statement

All institutional and national guidelines for the care and use of laboratory animals were followed and approved by the appropriate institutional committees (IRB Approval NO. 109086).
