# Supplementary material for: Syndecan-4 is required for early-stage repair responses during zebrafish heart regeneration
Source: Mol Biol Rep. 2024 May 3;51(1):604. doi: 10.1007/s11033-024-09531-4 (PMC11068835; doi:10.1007/s11033-024-09531-4)
Supplement: Supplementary file 1 — Supplementary file1 (DOCX 5892 KB) [file 11033_2024_9531_MOESM1_ESM.docx]

**Syndecan-4 is required for early-stage repair responses during zebrafish heart regeneration**

***Molecular Biology Reports***

Zih-Yin Lai^1,2^, Chung-Chi Yang^1,2,3,4^, Po-Hsun Chen^1,2^, Wei-Chen Chen^1,2^, Ting-Yu Lai^1,2^, Guan-Yun Lu^1,2^, Chiao-Yu Yang^1,2^, Ko-Ying Wang^1,2^, Wei-Cen Liu^1,2^, Yu-Chieh Chen^1,2^, Lawrence Yu-Min Liu^5,6^ and Yung-Jen Chuang^1,2^

^1^School of Medicine, National Tsing Hua University, Hsinchu 300044, Taiwan, R.O.C.

^2^Institute of Bioinformatics and Structural Biology, National Tsing Hua University, Hsinchu 300044, Taiwan, R.O.C.

^3^Division of Cardiovascular Medicine, Taoyuan Armed Forces General Hospital, Taoyuan City 325208, Taiwan, R.O.C.

^4^Cardiovascular Division, Tri-Service General Hospital, National Defense Medical Center, Taipei City 114201, Taiwan, R.O.C.

^5^Department of Internal Medicine, Division of Cardiology, Hsinchu MacKay Memorial Hospital, Hsinchu 300044, Taiwan, R.O.C.

^6^Department of Medicine, MacKay Medical College, New Taipei City 252005, Taiwan, R.O.C.

**Note:** Zih-Yin Lai and Chung-Chi Yang are co-first authors due to equal contributions of these two authors.

**Note:** Both Lawrence Yu-Min Liu and Yung-Jen Chuang are corresponding authors.

Main corresponding author:

Yung-Jen Chuang, Ph.D.

School of Medicine, National Tsing Hua University, Hsinchu 300044, Taiwan, R.O.C. &

Institute of Bioinformatics and Structural Biology, National Tsing Hua University, Hsinchu 300044, Taiwan, R.O.C.

E-mail: [yjchuang@life.nthu.edu.tw](mailto:yjchuang@life.nthu.edu.tw)

Tel: 886-3-5742764

Fax: 886-3-5715934

Co-corresponding author:

Lawrence Yu-Min Liu, M.D., Ph.D.

Department of Internal Medicine, Division of Cardiology, Hsinchu MacKay Memorial Hospital, Hsinchu 300044, Taiwan, R.O.C.

&

Department of Medicine, MacKay Medical College, New Taipei City 252005, Taiwan, R.O.C.

E-mail: [drlawrenceliu@gmail.com](mailto:drlawrenceliu@gmail.com)

Tel: 886-3-6119595

Fax: 886-3-6111175

**Supplementary Figures**

**
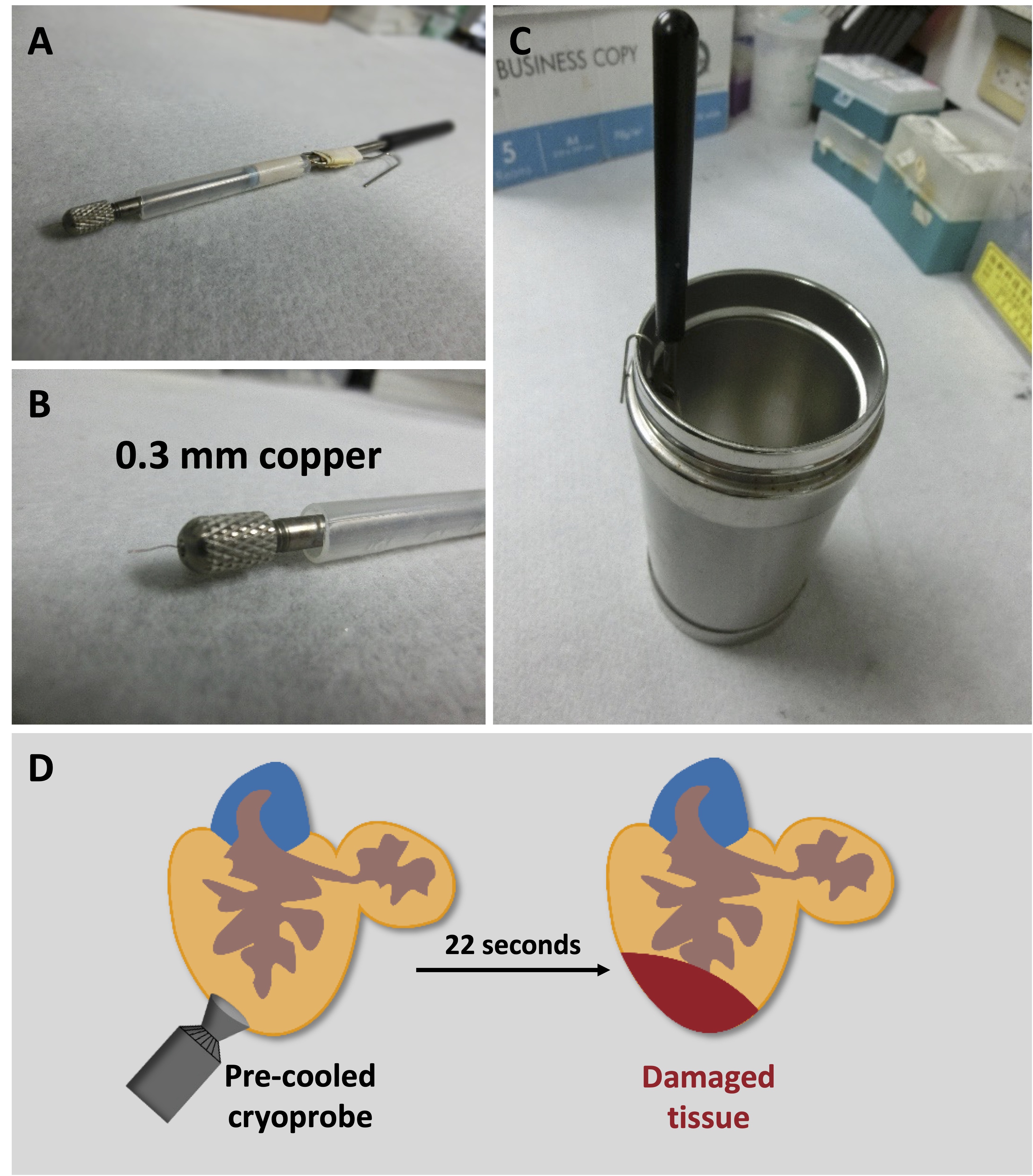
**

**Fig. S1 Cryoinjury apparatus and procedure for zebrafish.**

**(A)** The cryoprobe rod was encased in a plastic tube for insulation. **(B)** A copper filament (0.3 mm diameter) ran through the tip of the threaded rod. **(C)** The cryoprobe was pre-cooled in a liquid nitrogen-filled steel cylinder for at least 15 minutes. **(D)** Cryoinjury of the adult zebrafish ventricle. A small incision was made in the chest using forceps, and the pre-cooled cryoprobe was applied to the exposed ventricle for 22 seconds.

**
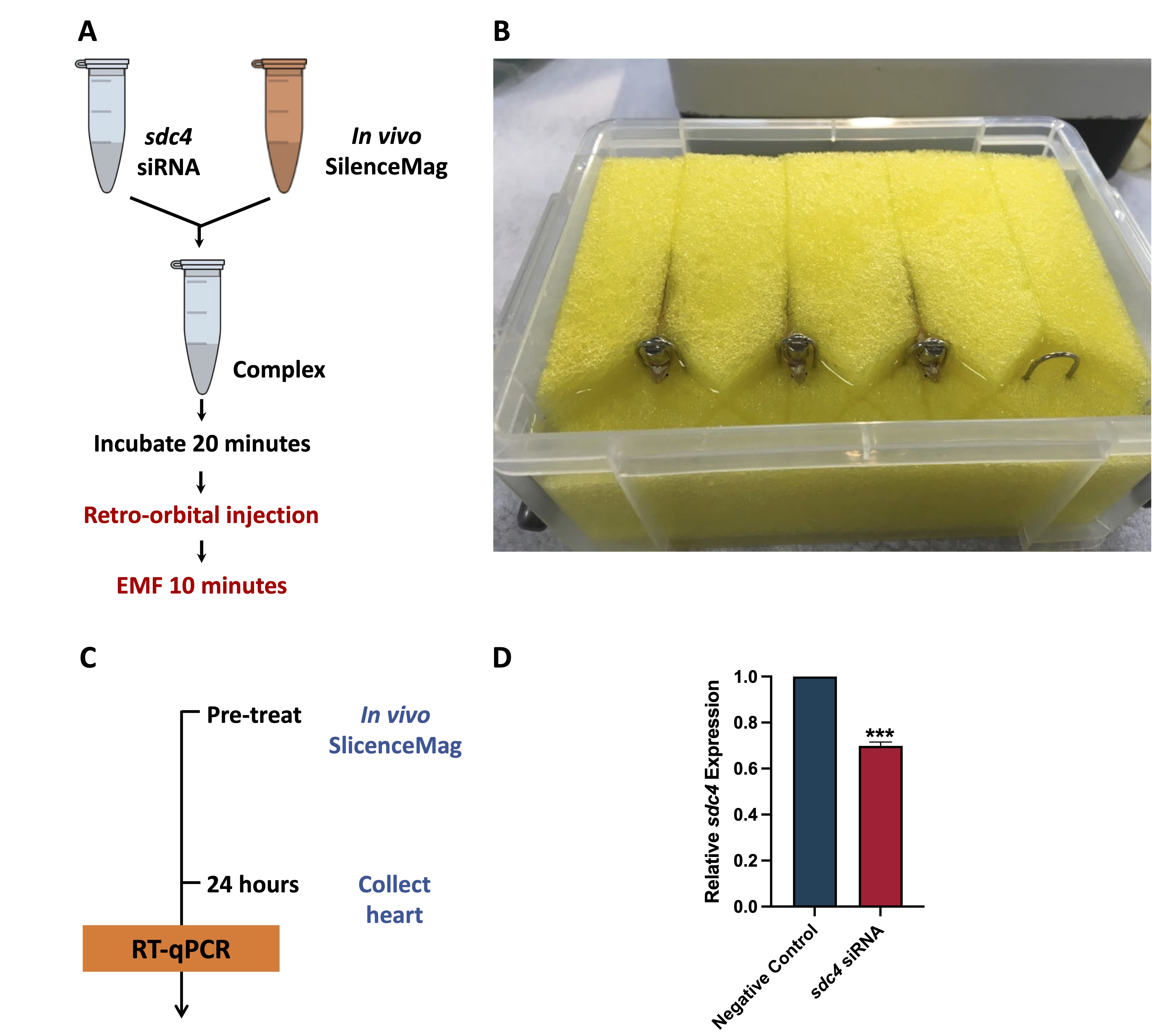
**

**Fig. S2 *sdc4* knockdown in zebrafish hearts using SilenceMag.**

**(A) *In* *vivo* SilenceMag procedure:** Zebrafish received retro-orbital injections of *sdc4* siRNA and SilenceMag followed by exposure to an external magnetic field (EMF). **(B)** Zebrafish were anesthetized and immobilized for heart exposure to the EMF for 10 minutes. **(C)** Schematic of the *sdc4* siRNA treatment plan and subsequent real-time quantitative PCR (RT-qPCR) analysis. **(D)** The *sdc4* gene expression in zebrafish hearts was significantly reduced by *in vivo* SilenceMag treatment compared to the negative control group (****p* < 0.005; n=8 per group).

**
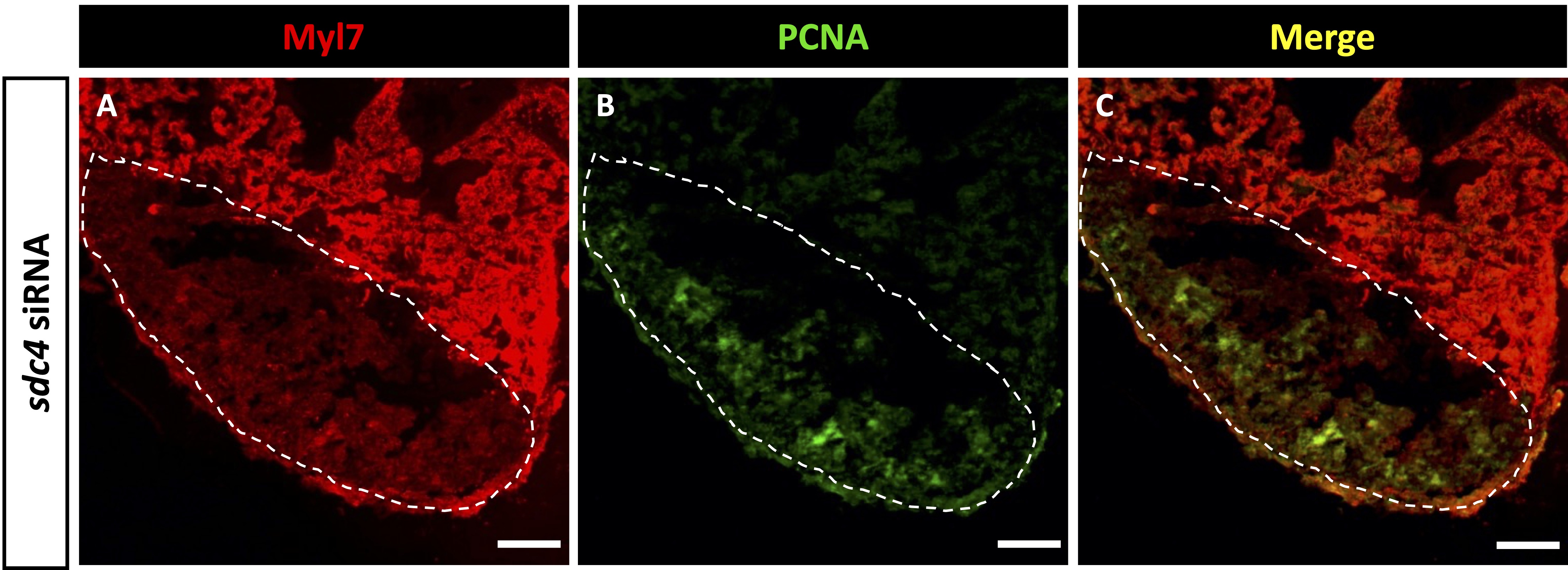
**

**Fig. S3 Absence of Myl7 and PCNA co-localization on 7 dpci at the injury site.**

Immunofluorescence staining of heart sections on 7 dpci was performed to assess the co-localization of Myl7 (cardiomyocyte marker) and PCNA (proliferation marker). Myl7 staining (red; GTX128346; GeneTex) is shown in panel **(A)**, PCNA staining (green) in panel **(B)**, and the merged image in panel **(C)**. The dashed line in all panels indicated the injury site. As evident in the merged image **(C)**, Myl7 expression did not co-localize with PCNA expression within the injury area, suggesting minimal cardiomyocyte proliferation at the site of injury. Scale bar: 100 μm.

**
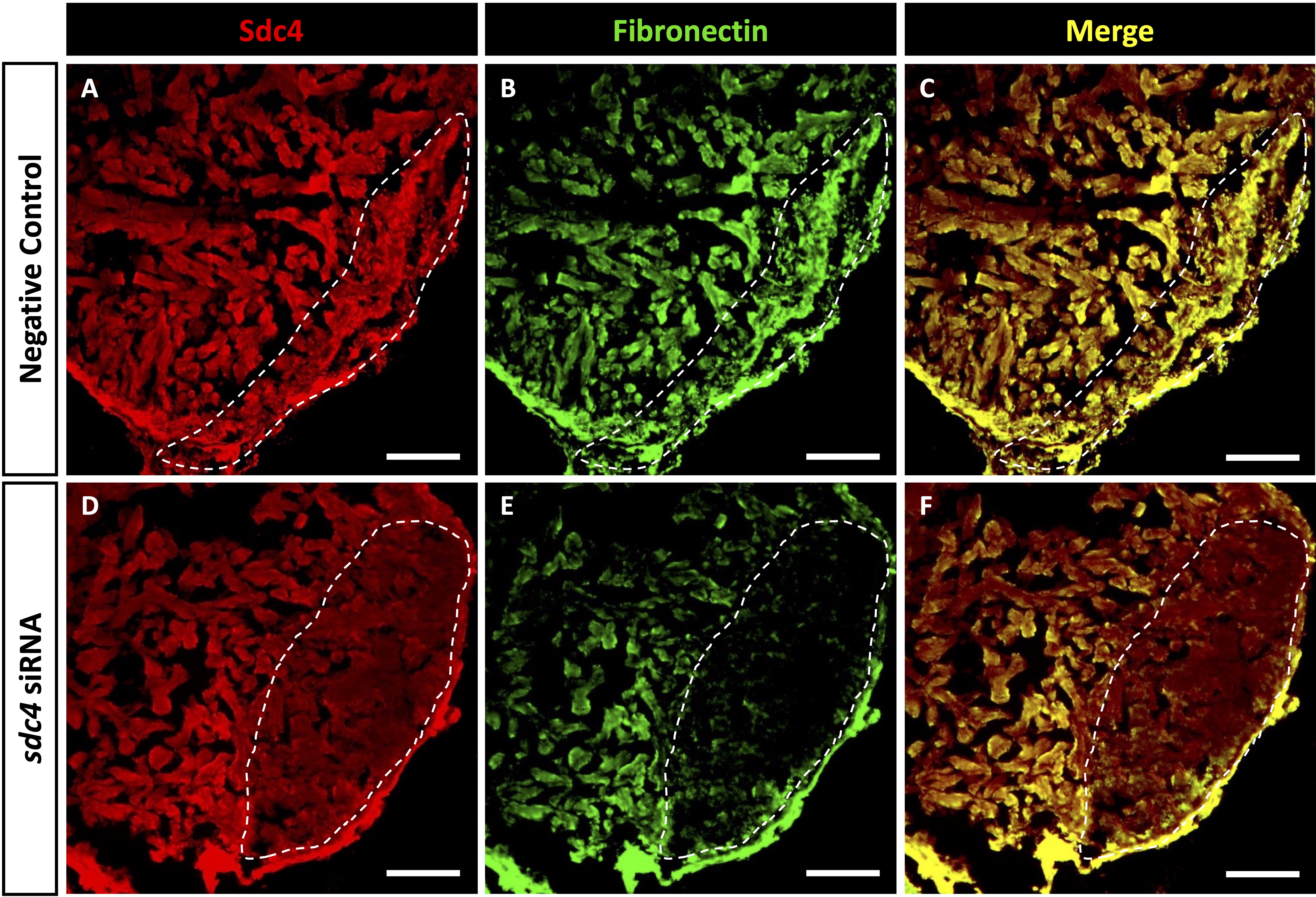
**

**Fig. S4 Sdc4 expression correlated with Fibronectin on 1 dpci at the cryoinjury site.**

Immunofluorescence staining of heart sections on 1 day after cryoinjury was performed to evaluate the co-localization of Sdc4 (syndecan-4) and Fibronectin. Panels **(A)** and **(D)** showed Sdc4 staining (red) in negative control and *sdc4* siRNA-treated groups, respectively. Panels **(B)** and **(E)** depicted Fibronectin staining (green) in the corresponding groups. The merged images in panels **(C)** and **(F)** demonstrated a high degree of co-localization between Sdc4 and Fibronectin at the injury site (dashed line) in both groups. Scale bar: 100 μm.

**Supplementary Tables**

**Table S1 The sequence of *sdc4* siRNA.**

| **siRNA** | **Sense (5’→3’)** | **Antisense (5’→3’)** |
| --- | --- | --- |
| *sdc4* siRNA | GCAGAUGUGAUCGAUGAUATT | UAUCAUCGAUCACAUCUGCTT |

**Table S2 The sequence of primers used for real-time quantitative PCR analysis.**

| **Gene** | **Forward (5’→3’)** | **Reverse (5’→3’)** |
| --- | --- | --- |
| *gapdh* | AATTCCTGAGCTCAATGGCAAG | AACCTGGTGCTCCGTGTATC |
| *sdc4* | CAAGGCCAACTGTCGATGAG | TTGATGCGGAGAACGAAGTG |
| *tgfb1a* | TCAAATGGCAACTCTCTTGC | AGTTGGCAAAGTACCCCTTG |
| *col1a1a* | GCTCCAAGCCTGAGGATGTC | GGTGTGCGACGTACAACCAT |
| *mmp2* | TGGATCCAGGCTTCCCTAAA | TTTTTGACCTCGCCGACTTT |
| *mmp9* | TGGAGTGCCCATTGATTCAC | TGAATGTCACTGCATTTCAGG |
